# 1019. Respiratory Syncytial Virus (RSV) Diagnoses in Hospitalized Patients Increases when Additional Specimen Types are Added to Nasopharyngeal Swab: Results from the RSV in Adults Multispecimen Study

**DOI:** 10.1093/ofid/ofad500.050

**Published:** 2023-11-27

**Authors:** Julio Ramirez, Ruth Carrico, Ashley M Wilde, Alan Junkins, Thomas R Chandler, Stephen Furmanek, Robin Hubler, Qing Liu, Sonal Uppal, Paula Peyrani, Negar Aliabadi, Bradford D Gessner, Elizabeth Begier

**Affiliations:** Norton Healthcare, Louisville, Kentucky; Norton Healthcare, Louisville, Kentucky; Norton Healthcare, Louisville, Kentucky; Norton Healthcare, Louisville, Kentucky; Norton Healthcare, Louisville, Kentucky; Norton Healthcare, Louisville, Kentucky; Pfizer Inc., Collegeville, Pennsylvania; Pfizer Inc., Collegeville, Pennsylvania; Pfizer, Champaign, Illinois; Pfizer, Inc, Collegeville, Pennsylvania; Pfizer, Champaign, Illinois; Pfizer Biopharma Group, Collegeville, Pennsylvania; Pfizer Vaccines, Dublin, Dublin, Ireland

## Abstract

**Background:**

RSV infection diagnosis in hospitalized adults is based primarily on PCR testing of nasopharyngeal (NP) swabs. Adding sputum is known to increase diagnostic yield, and saliva has been successfully used for viral respiratory infection diagnosis, however, no study has evaluated the synergistic effect of adding both sample types concurrently

We sought to define the increase in RSV prevalence when diagnosed by PCR of NP swab plus sputum and/or saliva versus NP swab PCR alone in adult patients hospitalized with acute respiratory illness (ARI) over two seasons, overall and by subgroup. First season overall findings were previously presented at ID week; this analysis adds season 2 data, allowing for subgroup analyses.

**Methods:**

This was a prospective cohort study of patients aged ≥40 years and hospitalized for ARI in four hospitals in Louisville, Kentucky during two seasons: 27Dec2021–01Apr2022 and 22Aug2022–03Mar2023. NP swab, saliva, and sputum specimens were obtained, and PCR tested with Luminex ARIES platform. RSV prevalence was calculated for diagnosis by NP swab alone, and NP swab plus sputum and/or saliva. Increase in RSV diagnosis with additional sample testing was compared for the whole cohort, for those with all three specimen types obtained, and by age, sex, and immune status.

**Results:**

We enrolled 2,798 patients and collected NP swabs in 100%, saliva in 99%, and sputum in 31% of patients. Among all patients, 133 (4.8%) had RSV diagnosed by any specimen versus 80 (2.9%) diagnosed by NP swab alone, consistent with an 66% increase in prevalence (i.e., 1.66-fold). Among 856 patients with three specimens obtained, 62 (7.2%) patients were diagnosed by any specimen, versus 37 (4.3%) patients with RSV diagnosed by NP swab alone. RSV detection trended higher among men (2.00), middle-aged adults (2.03; 40–65 years), and immunocompromised persons (1.78) (Table 1).

Table 1

**Figure:**
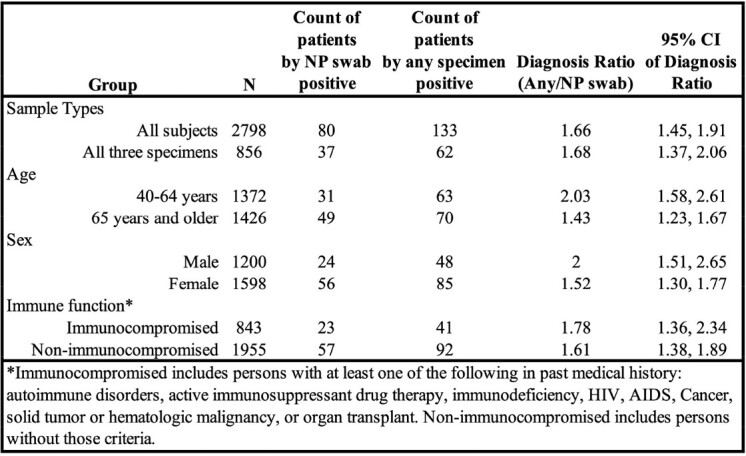

Increase in RSV detection associated with testing additional specimen types beyond NP swab for specific populations

**Conclusion:**

RSV infections were frequently detected in saliva and sputum samples. Standard-of-care PCR testing of NP swabs for RSV underestimates RSV prevalence in adult patients hospitalized with ARI. Hospitalized RSV ARI burden estimates in adults based solely on NP swab RT-PCR should be adjusted for underestimation.

**Disclosures:**

**Ruth Carrico, PhD, DNP, APRN**, Moderna: COVID-19 vaccine|Pfizer: Grant/Research Support|Pfizer: PCV20|Pfizer: Meningococcal and Pneumococcal vaccines; Paxlovid|Sanofi: Speaker; influenza vaccine|Seqirus: Influenza vaccine|Valneva: Travel vaccines **Ashley M. Wilde, PharmD, BCIDP**, Pfizer: Grant/Research Support **Robin Hubler, MS**, Pfizer: Employee|Pfizer: Stocks/Bonds **Qing Liu, M.S.**, Pfizer Inc.: Stocks/Bonds **Paula Peyrani, MD**, Pfizer, Inc: Employee|Pfizer, Inc: Stocks/Bonds **Negar Aliabadi, MD, MS**, Pfizer: Employee **Bradford D. Gessner, M.D, M.P.H.**, Pfizer: I am an employee of Pfizer|Pfizer: Stocks/Bonds **Elizabeth Begier, M.D, M.P.H.**, Pfizer: EB is an employee of Pfizer, the sponsor of this study|Pfizer: Stocks/Bonds

